# Outbreak-associated *Vibrio cholerae* Genotypes with Identical Pulsotypes, Malaysia, 2009

**DOI:** 10.3201/eid1807.111656

**Published:** 2012-07

**Authors:** Cindy Shuan Ju Teh, Zarizal Suhaili, King Ting Lim, Muhamad Afif Khamaruddin, Fariha Yahya, Mohd Hailmi Sajili, Chew Chieng Yeo, Kwai Lin Thong

**Affiliations:** University of Malaya, Kuala Lumpur, Malaysia (C.S.J. Teh, K.T. Lim, K.L. Thong);; and Universiti Sultan Zainal Abidin, Kuala Terengganu, Malaysia (Z. Suhaili, M.A. Khamaruddin, F. Yahya, M.H. Sajili, C.C. Yeo)

**Keywords:** bacteria, antibacterial, antimicrobial resistance, cholera outbreak, *ctxB*, PFGE, *Vibrio cholerae*, Malaysia

## Abstract

A cholera outbreak in Terengganu, Malaysia, in November 2009 was caused by 2 El Tor *Vibrio cholerae* variants resistant to typical antimicrobial drugs. Evidence of replacement of treatable *V. cholerae* infection in the region with antimicrobial-resistant strains calls for increased surveillance and prevention measures.

*Vibrio cholerae*, the causative agent of cholera, is endemic in many parts of the world, especially in countries that lack clean water supplies and adequate public health facilities (*1*). In Malaysia, cholera outbreaks caused by the El Tor O1 *V. cholerae* serogroup occur periodically, cases from the 0139 serogroup occur sporadically, and the non–O1/non–O139 *V. cholerae* serogroup has not been implicated in any major outbreak (*2**–**4*). Contaminated drinking water, cooked food, and raw or undercooked seafood served as vehicles of transmission in Malaysia (*5*).

## The Study

In November 2009, a cholera outbreak occurred in Terengganu, Peninsular Malaysia. The outbreak began in the capital, Kuala Terengganu, and spread to several districts within a week. Approximately 400 persons were hospitalized for treatment of acute diarrhea and its complications during the outbreak period. One death occurred before the local health authorities declared an outbreak. Five ice factories, 2 fish cracker factories, and several restaurants and street cart food vendors were ordered closed because they were suspected of being possible sources of the outbreak (Ministry of Health, Malaysia, unpub. data).

For this study, 75 rectal swab samples, collected from patients admitted to Hospital Sultanah Nur Zahirah in Kuala Terengganu who had acute diarrhea during the outbreak period, were available for analysis. In addition, 60 environmental samples (6 water samples, 54 environmental swab samples) were collected from 2 of the ice factories (factories A and B) in Kuala Terengganu by the Terengganu State Department of Health during the outbreak period and were provided to us for analysis. Environmental swab samples were obtained from several areas within the ice-making factories. The rectal swab and environmental samples were enriched overnight in alkaline-buffered peptone water, pH 8.6 (Oxoid, Basingstoke, UK) and cultured on thiosulfate citrate-bile salts-sucrose agar (Oxoid). The presumptive colonies were subjected to conventional biochemical tests, such as string, salt tolerance, Voges-Proskauer, lysine iron agar, Kliger iron agar, and arginine dihydrolase testing. PCRs targeting *ompW*, *hlyA*, *rfb*, *ctxA*, *toxR*, *tcpI*, *rtxC*, *rstR*, and *tcpA* genes as described (*6**,**7*) were run in parallel to confirm and characterize *V. cholerae* isolates. Template DNA was also prepared directly from the water samples as described (*6*) for detection of viable but nonculturable *V. cholerae* and its virulence genes.

Antimicrobial drug susceptibility of the confirmed *V. cholerae* isolates was determined by the disk diffusion method according to Clinical and Laboratory Standards Institute guidelines (*8*). Six antimicrobial agents (Oxoid) were used: ampicillin (10 µg), chloramphenicol (30 µg), ciprofloxacin (5 µg), trimethoprim/sulfamethoxazole (25 µg), erythromycin (15 µg), and tetracycline (30 µg). To determine the genetic relatedness of the isolates, pulsed-field gel electrophoresis (PFGE) was performed according to the established PulseNet protocol (*9*) and analyzed with BioNumerics 6.0 (Applied Maths, Kortrijk, Belgium); *ctxB* genotyping was also performed as described (*10*).

On the basis of conventional biochemical tests and PCR, 37 isolates from the rectal swab samples and 1 isolate from the washroom swab sample of ice factory B were confirmed as *V. cholerae,* showing an isolation rate of 48.0% for the clinical samples and 1.9% for the environmental samples. In addition, the 37 clinical isolates were identified as El Tor O1 on the basis of Voges-Proskauer tests and were positive for *hlyA*^El^, *tcpA*^El^, *rstR*^El^, *rtxC*, and *rfb*O1 genes. The *ctxA*, *toxR*, and *tcpI* genes were present in all of the clinical isolates. The isolate from the restroom specimen of factory B was identified as a non–O1/non–O139 *V. cholerae* strain that had *hlyA*^El^, *rstR*^El^, and *toxR* genes. This finding indicated that this isolate was likely not related to the outbreak in question. No amplification of *V. cholerae*–specific genes was observed for the DNA extracted directly from the water samples.

Although the environmental non-O1/non-O139 *V. cholerae* isolate was sensitive to all the antimicrobial agents tested, the 37 clinical O1 *V. cholerae* isolates were resistant to ampicillin, trimethoprim/sulfamethoxazole, erythromycin, and tetracycline. In Malaysia, tetracycline generally has been considered the drug of choice for cholera treatment; however, it has been replaced by erythromycin because the number of tetracycline-resistant strains has increased since a 1992 outbreak in the state of Kelantan (*11*). The emergence of erythromycin-resistant isolates in this outbreak will likely contribute to decreased efficacy of erythromycin.

**Figure 1:**
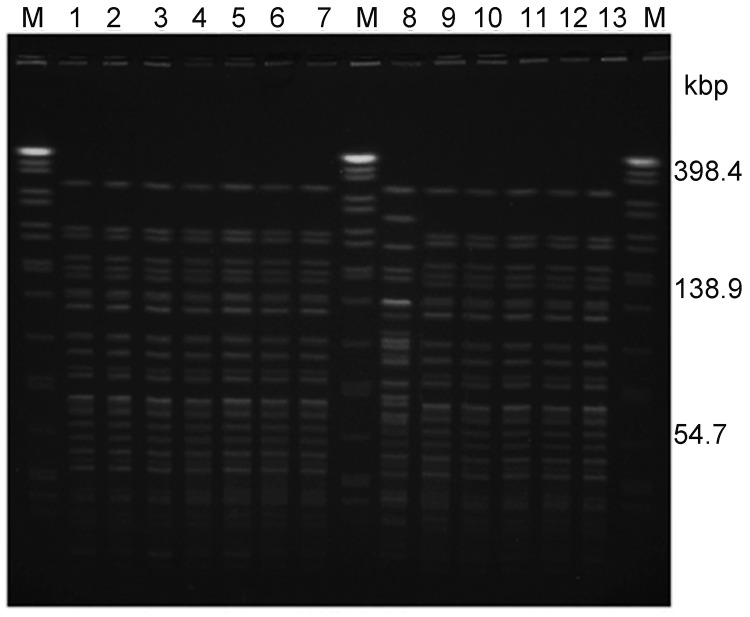
*Not*I-pulsed-field gel electrophoresis profiles of *Vibrio cholerae* isolated during the outbreak, Terengganu, Malaysia, 2009. Lane M: *Xba*I-digested *Salmonella enterica* serovar Braenderup H9812 as DNA standard; lanes 1–7 and 9–12: isolates of El Tor O1 serogroup (rectal swab); lane 8: isolate of non–O1/non–O139 serogroup (swab from ice factory); lane 13: El Tor O1 *V. cholerae* isolated in 2008 (Kuala Lumpur).

PFGE of *Not*I-digested chromosomal DNA from all of the human isolates resulted in 1 pulsotype with 24 fragments (≈30 kb to ≈370 kb); the environmental isolate showed a distinct pulsotype (*F* = 0.83) (Figure 1). PFGE was repeated 2× with identical results. In addition, the pulsotype of the clinical isolates in this study was identical to the pulsotype of an O1 isolate (123/08) from a cholera patient in Kuala Lumpur in 2008 (Figure 1) (*12*). Isolate 123/08 also showed identical antibiograms with the Terengganu O1 outbreak isolates. This finding suggests that isolate 123/08 and the Terengganu 2009 O1 outbreak isolates were possibly linked.

**Figure 2:**
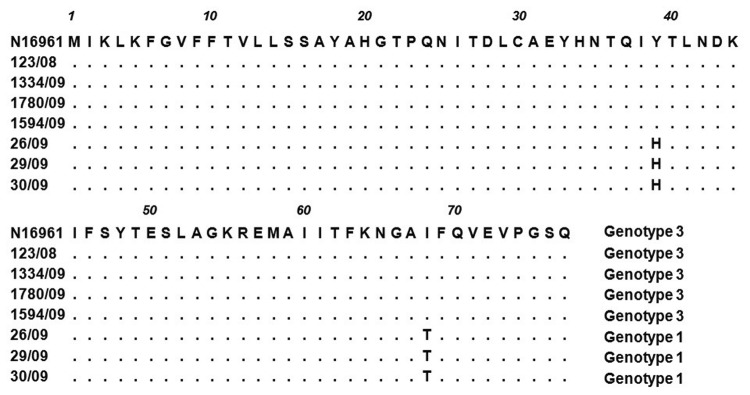
Amino acid sequence alignment of the *ctxB* subunit of representative *Vibrio cholerae* isolates from the cholera outbreak, Terengganu, Malaysia, 2009. El Tor O1 N16961 (*ctxB3*) was used as the reference strain in the alignment. Identical amino acid residues are indicated by dots. Two genotypes (*1**,**3*) were observed in the outbreak strains.

Several *ctxB* alleles have been identified among O1 *V. cholerae* strains on the basis of a few point mutations: 1) classical and El Tor, US Gulf Coast (39His, 46Phe, 68Tyr); 2) El Tor, Australia (39His, 46Leu, 68Tyr); and 3) El Tor, seventh pandemic, and El Tor, Latin American epidemic (39Tyr, 46Phe, 68Ile) (*10*). Of the 37 isolates, 33 were classified as genotype 3 on the basis of multiple sequence alignments of *ctxB* (*10*). Similarly, our previous multilocus sequence typing study (*2*) subtyped isolate 123/08 as an El Tor biotype characterized by the *ctxB3* gene (Figure 2). We postulate that this particular clone has reemerged since its isolation in Kuala Lumpur in 2008 and was likely linked to the 2009 Terengganu outbreak.

Four of the 37 clinical isolates showed amino acid substitutions Tyr39His and Ile68Thr (Figure 2) and were classified as genotype 1. Genotype 1 strains that carry both classical and El Tor *rstR* allele have been detected in Asian and African countries since the 1990s (*13*). In 2001, El Tor variant strains that harbored *rstR*^El^/*ctxB1* superseded the typical El Tor strains in Bangladesh and other Asian countries. Since 2007, outbreaks in Vietnam and Thailand have been mainly caused by El Tor variant strains (*7**,**14*). Ang et al. (*3*) reported that El Tor variant strains could have emerged earlier in Malaysia because 1 such strain was responsible for the 2000 outbreak in Kelantan. In contrast to neighboring countries, our current study indicates that the El Tor strains have not been replaced by El Tor variant strains in Malaysia; typical El Tor strains are still found in this country. In our current study, the isolates could not be differentiated by PFGE, although they belonged to 2 *ctxB* genotypes. This finding might be attributed to the genetic events resulting in the transfer of the different *ctxB* alleles among the *V. cholerae* populations in Malaysia or with the strains from neighboring countries.

## Conclusions

The 2009 cholera outbreak in Terengganu was controlled in late November; 187 cases and 1 death were confirmed (www.myhealth.gov.my/myhealth). We found no evidence of an association between 2 ice factories considered as possible sources of infection and the outbreak. The health authorities later ruled out the other ice factories, fish cracker factories, and eateries suspected of being sources and were unable to trace the source of the outbreak.

Two genotypes (ctxB1 and ctxB3) of the El Tor O1 *V. cholerae* serogroup with identical pulsotypes were likely responsible for the cholera outbreak in Terengganu in late 2009. Our findings support the need for increased surveillance in the region to document the prevalence of such strains. Preventive activities such as water sanitation, public education on proper food handling, and personal cleanliness are crucial to reduce the risk of spread of cholera.

## References

[1] Mandomando I, Espasa M, Valles X, Sacarlal J, Sigauque B, Ruiz J, Antimicrobial resistance of *Vibrio cholerae* O1 serotype Ogawa isolated in Manhica District Hospital, southern Mozambique. J Antimicrob Chemother. 2007;60:662–4. 10.1093/jac/dkm257 17626024

[2] Teh CSJ, Chua KH, Thong KL. Genetic variation analysis of *Vibrio cholerae* using multilocus sequencing typing and multi-virulence locus sequencing typing. Infect Genet Evol. 2011;11:1121–8. 10.1016/j.meegid.2011.04.005 21511055

[3] Ang GY, Yu CY, Balqis K, Elina HT, Azura H, Hani MH, Molecular evidence of cholera outbreak caused by a toxigenic *Vibrio cholerae* O1 El tor variant strain in Kelantan, Malaysia. J Clin Microbiol. 2010;48:3963–9. 10.1128/JCM.01086-10 20826646 PMC3020861

[4] Chen CH, Shimada T, Elhadi N, Radu S, Nishibuchi M. Phenotypic and genotypic characteristics and epidemiological significance of *ctx*+ strains of *Vibrio cholerae* isolated from seafood in Malaysia. Appl Environ Microbiol. 2004;70:1964–72. 10.1128/AEM.70.4.1964-1972.2004 15066786 PMC383156

[5] Lim VK. Cholera: a re-emerging infection. Med J Malaysia. 2001;56:1–3. 11503284

[6] Teh CSJ, Thong KL, Ngoi ST, Ahmad N, Nair GB, Ramamurthy T. Molecular characterization of serogrouping and virulence genes of Malaysian *Vibrio cholerae* isolated from different sources. J Gen Appl Microbiol. 2009;55:419–25. 10.2323/jgam.55.419 20118606

[7] Okada K, Roobthaisong A, Nakagawa I, Hamada S, Chantaroj S. Genotypic and PFGE/MLVA analyses of *Vibrio cholerae* O1: geographical spread and temporal changes during the 2007–2010 cholera outbreaks in Thailand. PLoS ONE. 2012;7:e30863. 10.1371/journal.pone.0030863 22292065 PMC3265523

[8] Clinical and Laboratory Standards Institute (CLSI). Performance standards for antimicrobial susceptibility testing. 18th informational supplement. CLSI document M100–S18. Wayne (PA): The Institute; 2008.

[9] Centers for Disease Control and Prevention. Pulsenet USA. The national molecular subtyping network for foodborne disease surveillance: rapid standard laboratory protocol for molecular subtyping of *Vibrio cholerae* by pulse-field gel electrophoresis (PFGE). 2006.

[10] Olsvik O, Wahlberg J, Petterson B, Uhlen M, Popovic T, Wachsmuth IK, Use of automated sequencing of polymerase chain reaction–generated amplicons to identify three types of cholera toxin subunit B in *Vibrio cholerae* O1 strains. J Clin Microbiol. 1993;31:22–5. 7678018 10.1128/jcm.31.1.22-25.1993 PMC262614

[11] Ranjit K, Nurahan M. Tetracycline resistant cholera in Kelantan. Med J Malaysia. 2000;55:143–5. 11072502

[12] Teh CSJ, Chua KH, Thong KL. Multiple-locus variable-number tandem repeat analysis of Vibrio cholerae in comparison with pulsed field gel electrophoresis and virulotyping. J Biomed Biotechnol. 2010;2010:817190. 10.1155/2010/817190 20671932 PMC2910556

[13] Safa A, Nair GB, Kong RY. Evolution of new variants of *Vibrio cholerae* O1. Trends Microbiol. 2010;18:46–54. 10.1016/j.tim.2009.10.003 19942436

[14] Tran HD, Alam M, Trung NV, Van Kinh N, Nguyen HH, Pham VC, Multi-drug resistant *Vibrio cholerae* O1 variant El Tor isolated in northern Vietnam between 2007 and 2010. J Med Microbiol. 2012;61:431–7. 10.1099/jmm.0.034744-0 22016560 PMC3347965

